# The Dark Side Effect of Entrepreneurial Resilience Diversity on Pivoting: The Role of Team Reflexivity

**DOI:** 10.3390/bs13110899

**Published:** 2023-10-30

**Authors:** Jialiang Fu, Renhong Zhu, Qin Liu, Yishuo Jiao, Xiaowei Li

**Affiliations:** School of Business, Sun Yat-sen University, Guangzhou 510275, China

**Keywords:** entrepreneurial team resilience diversity, pivoting, team reflexivity, sensemaking theory, environmental hostility

## Abstract

Resilience is widely recognized as a form of psychological capital that helps entrepreneurs cope with challenges in the face of adversity by actively adjusting business strategies. Prior research has investigated the effect of resilience on pivoting, which is an important entrepreneurial decision to forgo the original business opportunity and exploit new opportunities. Despite the increasing empirical evidence on the effect of resilience on strategic changes and the pivot, the literature may have overstated the benefits of entrepreneurial resilience while neglecting its potential dark sides. Hence, the current study focuses on the structure of resilience within an entrepreneurial team and introduces the concept of resilience diversity. Drawing from sensemaking theory, we develop a research framework that investigates the dark-side effects of resilience diversity on team reflexivity and pivoting and the moderating effect of environmental hostility. Empirical results from a two-wave survey of 112 entrepreneurial teams in China reveal that resilience diversity negatively affects pivoting by impairing the quality of team reflexivity. Moreover, the mediating effect of team reflexivity is strengthened in hostile environments. These findings contribute to the literature on entrepreneurial resilience, pivot, and team reflexivity, and provide important practical implications for entrepreneurial teams.

## 1. Introduction

During their business journey, entrepreneurs may frequently encounter various setbacks and adversities [1]. In the face of the political, economic, technological or cultural changes [2], entrepreneurs’ capabilities to conduct pivoting activities and entrepreneurs’ activities of radical adjustment in entrepreneurial opportunities [3] are crucial to the survival and growth of new ventures [4,5]. Based on the concept of resilience in psychology, entrepreneurial resilience denotes entrepreneurs’ capability to effectively recover from negative emotions under pressure [6], adjust their business strategies to cope with setbacks [7], and even achieve growth through learning activities [8]. Therefore, as one of the fundamental components of entrepreneurship, entrepreneurial resilience has gained significant attention in entrepreneurship research [9].

Although adversity often brings challenges to entrepreneurs, it also leads to the emergence and extinction of entrepreneurial opportunities [10]. Therefore, entrepreneurs often need to promptly adjust their original opportunities to cope with the ever-changing and dynamic environments. As a commonly used coping strategy in adversity [11], pivoting has emerged as the most prevalent and vital decision in entrepreneurship. Since pivoting enables entrepreneurs to overcome the current crisis and significantly impact entrepreneurial survival and success [12], whether resilience enables entrepreneurs to better enact pivoting in response to setbacks has become an important topic in the current research stream.

Existing research mainly focuses on the positive effect of entrepreneurial resilience. Resilient entrepreneurs are more confident with a lower fear of unknown risks, and more willing to regard exploratory innovation activities as opportunities rather than risky events [13], which promotes creativity, innovation, and therefore pivoting. Moreover, some studies indicate that resilient entrepreneurs are better equipped to seize new opportunities that are distinct from the original ones [14] to enact the pivot [15] and strategic changes [16,17] in adversities.

However, while existing studies primarily investigate the positive impact of resilience on pivoting, some researchers have challenged the widely accepted assumption that entrepreneurial resilience is universally beneficial. For instance, studies on the potential dark side of resilience have provided initial evidence that high resilience may lead to over-optimistic self-concepts [18] and the excessive consumption of resources [19], which may be detrimental to pivoting. Also, in practice, despite the significance of the pivot in adversities, some resilient entrepreneurs often choose not to pivot. Therefore, the mixed and contradictory relationship between resilience and pivoting underlines a core conflict in the resilience literature: Is resilience beneficial or detrimental? Due to the importance of pivoting, in this study, we focus on a more detailed and accurate question: Will higher entrepreneurial resilience necessarily lead to better enactment of the pivot? By doing so, we attempt to supplement the existing divergent results.

While limited research has partially revealed the negative effects of entrepreneurial resilience on pivoting, two critical research gaps still exist when team entrepreneurship becomes increasingly prevalent. First, most entrepreneurial firms are established and managed by teams instead of single individuals [20], and the pivoting decisions are usually undertaken by the entrepreneurial team [5]. Since the formation of high-quality pivoting decisions requires entrepreneurial teams to collectively scrutinize the environment and reach a consensus, team interaction and cognitive processes may profoundly affect the quality of pivoting decisions. However, prior research has neglected the cooperative nature of many new ventures and overlooked the peer effect of the resilience level of team members. It is essential to examine the peer effect of team members’ resilience to adequately capture the impact of resilience on pivoting in the team context. Second, the research stream of team resilience primarily focuses on the average level of resilience within a team, assuming that team members are similar in resilience to some extent [21], which overlooks the existence of variation and dispersion in resilience within teams. This limited focus restricts our comprehension of a crucial aspect of resilience that manifests only in entrepreneurial teams, i.e., resilience diversity. Given that team characteristics encompass average and variation levels, resilience in a team may exhibit significant individual differences [22], which may strongly influence the process of cooperation and decision making [23]. Research in team diversity provides initial evidence that trait diversity may increase emotional conflict [24], which may be detrimental to pivoting activities. The limited focus on resilience diversity is surprising and therefore worth studying.

Hence, in the context of team entrepreneurship, we focus on the structure of resilience in teams by introducing the concept of resilience diversity. We propose that it is essential to consider the disparity of resilience among team members because resilience diversity may have a dark side effect on pivoting. Moreover, since the pivot increases the complexity of the team’s decision-making process [10], entrepreneurial teams need to frequently reflect on their goals, strategies, and procedures [25]. The dual focus of team reflexivity [26] requires teams to recognize the gap between the actual and expected outcomes of the original opportunity under environmental changes [27], and to gather and interpret information comprehensively to identify novel opportunities for future actions. Consequently, the dual focus of team reflexivity is consistent with the two perspectives of pivoting, i.e., questioning the original opportunity and exploiting new ones [12]. As such, team reflexivity may mediate the relationship between team diversity and pivoting. Furthermore, when explaining the mediating effects of team reflexivity, the sensemaking theory offers an appropriate theoretical lens. The collaborative effort of the pivot decision is a fundamentally team-level, ongoing process of sensemaking [3], and entrepreneurial resilience also represents a psychological framework of values and thinking patterns amid adversity, thus reflecting a process of making sense of the environment and tasks [22]. Therefore, based on sensemaking theory, we posit that the fundamental prerequisite for pivoting is that entrepreneurial teams rationally analyze and respond to the performance of the original opportunity, and make sense of the information from the feedback to discover new opportunities. As a sensemaking process [28], team reflexivity may be influenced by team diversity and may further impact the adjustment and updates of the teams’ cognitive frameworks, thereby influencing the team’s pivot decisions.

Furthermore, the impacts of team diversity are highly contingent [29]. Entrepreneurial resilience is a trait that enables entrepreneurs to cope with adversity, while the objective of team reflexivity is to align with the external dynamic environment [27]. Therefore, the characteristics of the external environment may function as boundary conditions. Environmental hostility may influence not only the function of entrepreneurial resilience among entrepreneurs [30] but also whether teams can successfully engage in reflection activities [31]. Thus, in this study, we examine the moderating effect of environmental hostility.

This study makes several significant theoretical contributions to the literature on entrepreneurial resilience, pivoting, and team reflexivity. First, this study allows us to form a more balanced understanding of entrepreneurial resilience. By connecting the literature on resilience and team diversity, we challenge the prevailing belief that resilience is always beneficial and positive and respond to the call of Hartmann et al. [32] and Williams et al. [9] to examine its potential dark sides. By focusing on the unclear relationship between resilience and pivoting, this study provides a comprehensive understanding of entrepreneurial resilience by supplementing a vital property of resilience that only manifests in the team context, i.e., resilience diversity. This study provides new insights showing that the introduction of highly resilient entrepreneurs into teams is not always beneficial, because it may increase resilience diversity with many low-resilience members and be detrimental to team reflexivity and pivoting. Second, we complement the literature on pivoting by introducing the entrepreneurial resilience diversity into the literature on pivoting and conducting empirical analysis. Existing studies mainly examine the external antecedents of pivoting, including the environmental characteristics and stakeholder feedback [3]. Empirical research on internal factors (such as resilience) is still insufficient. This study bridges the gap by empirically examining the impact of resilience diversity as an internal factor. In doing so, this exploration provides an important research perspective of pivoting, and deepens our understanding of the antecedents. Third, the current study extends the literature on team reflexivity. Prior research on team reflexivity has primarily examined how reflexivity is affected by the functional characteristics of a team [31] and overlooked the role of social diversity. We respond to Humphrey and Aime’s [33] call for exploring the impact of individual traits and their dispersion on team reflexivity by focusing on resilience and its diversity. Furthermore, this study connects team reflexivity with pivoting and investigates the impact of this critical collective cognitive process on pivot decisions. These explorations enrich the research perspective of team reflexivity and pivoting, and increase our understanding of how pivot decisions are undertaken in diversified teams.

Following the introduction, the remaining sections of this paper are as follows: Section 2 presents the literature review, the hypothesis development and the theoretical model. Section 3 introduces the research methodology, and Section 4 shows the results and findings of this study. Section 5 discusses the results, including theoretical implications, practical implications, limitations, and conclusion.

## 2. Theory and Hypotheses

In this study, we develop our theory and hypotheses based on sensemaking theory. Sensemaking theory provides a valuable perspective to comprehend organizational and team processes [34]. It posits that social reality depends on individuals’ cognition processes and sensemaking activities [35]. Differences or conflicts are at the core of initiating and maintaining the sensemaking process. When different clues interrupt an individual’s ongoing activities, sensemaking activities may occur [36]. Sensemaking activities refer to the use of environmental cues to construct an understanding of novel or puzzling events and the meaning-creation process through a cycle of internal interpretation and external action [37]. Through the sensemaking process, individuals are able to form interpretations of the environment, which helps them effectively cope with uncertainty and ambiguity [27].

We propose that the theory of sensemaking provides a suitable framework for explaining the impact of resilience diversity on team reflexivity and thus pivoting. Entrepreneurial resilience is a psychological framework that represents individuals’ values, cognition patterns, and understanding of the tasks in adversity [22]. Therefore, resilience may influence the team’s interpretation of and response to adversity [7] and shape a shared and coherent sensemaking process at the team level. Furthermore, team reflexivity is also a process of sensemaking to adjust and update the team’s cognitive frameworks about the environment and tasks [28]. Also, the pivot decision made by entrepreneurial teams is a fundamentally team-level, ongoing process of sensemaking [3], which needs the collaborative effort of making sense of the environment and tasks by the whole team [22]. Therefore, based on sensemaking theory, we posit that resilience diversity may impact team reflexivity, the process of adjustment and updates of the teams’ cognitive frameworks. Furthermore, the fundamental prerequisite for pivoting is team reflexivity, a process of entrepreneurial teams to rationally analyze and respond to the performance of the original opportunity, and make sense of the information from the feedback to discover new opportunities.

### 2.1. Entrepreneurial Resilience and Its Dark Side Effects

The entrepreneurial process is frequently accompanied by adversities and challenges, thus requiring entrepreneurs to be resilient to promptly adjust their goals and strategies to cope with continuously emerging unexpected events [22,38]. Entrepreneurial resilience refers to entrepreneurs’ ability to effectively overcome threats in the short run and obtain sustained growth in the long run [39]. As a multidimensional concept, entrepreneurial resilience comprises three key subconcepts: recovering ability [32], coping ability [7], and thriving ability [40]. Since some actions of entrepreneurs are strongly driven by emotional mechanisms [41], emotions are crucial in the resilience process. The recovering ability is the capability of managing adverse situations by regulating negative emotions and maintaining a positive psychological state through self-regulation or external support [22]. The coping ability emphasizes entrepreneurs’ capability to actively respond to obstacles and skillfully mitigate crises [42], which allows entrepreneurs to tackle challenges head-on and find innovative solutions to navigate difficult circumstances. Meanwhile, the thriving ability underscores the significance of the learning activity during adversities. It turns challenges into valuable learning experiences and contributes to long-term growth and success [39]. These subconcepts collectively empower entrepreneurs to handle adversities emotionally and cognitively and grasp growth opportunities to thrive.

The increasing prevalence of team entrepreneurship has aroused researchers’ focus on team resilience [43]. As entrepreneurial teams often encounter challenges and uncertainty, understanding how teams collectively cope with and grow from adversities becomes crucial for their long-term success and sustainability [44]. Team resilience is the collective ability of a team to recover from adversities during crises, and the ability to adapt to and actively learn from challenges after crises [45]. Despite its significance, the literature on team resilience remains relatively limited compared with research on individual and organizational resilience [21].

Resilience is generally considered a form of psychological capital that benefits individuals [32]. Prior research on entrepreneurial resilience predominantly focuses on its positive effect by exploring how it enables entrepreneurs to cope with challenges effectively and leads to entrepreneurial success. Entrepreneurial resilience has been proven to be beneficial to learning activities [7,22], subjective well-being [46], and overall entrepreneurial performance [47]. Furthermore, introducing highly resilient team members increases the average level of team resilience and enables the entrepreneurial team to manage stress and share knowledge. Thus, the addition of these members improves overall team cooperation and entrepreneurial performance [43].

While the benefits of resilience have been widely acknowledged, its dark side has received limited attention and needs theoretical and empirical investigation [32]. First, resilience has been found to exhibit strong correlations with narcissism, which may lead to negative impressions from other actors [9] and complicate team collaboration. Furthermore, the resilience displayed amid adversity can generate considerable pressure and stress, which may harm entrepreneurs’ psychological well-being [48]. Second, resilient entrepreneurs often exhibit optimistic self-conceptions, which may hinder their ability to learn from adversities [18]. Moreover, resilience may discourage entrepreneurs from considering entrepreneurial exit as a favorable alternative in certain circumstances [9]. Failing to embrace “intelligent failure” can obstruct the adjustment of cognitive frameworks and the accumulation of knowledge [49]. As a result, this deficiency in failure learning may harm the quality of subsequent entrepreneurial decisions and lead to entrepreneurial failure [18].

Although some studies have explored the negative effects of entrepreneurial resilience, several gaps in the research need further investigation. First, prior studies have mainly focused on the impacts of resilience on entrepreneurs themselves rather than other actors. In entrepreneurial teams, members often interact with each other and may be influenced by other members’ traits and behaviors [43]. The peer effect of other team members’ resilience lacks research attention. Second, the majority of research on entrepreneurial team resilience has predominantly focused on the team’s average level of resilience, which assumes the homogeneity of members’ resilience [21]. Given that the characteristics of the psychological traits within a team include the average level and dispersion level [24], the structural differences in resilience in entrepreneurial teams need to be considered. Third, previous studies have mainly investigated the effects of entrepreneurial resilience on the recognition and exploitation of the original opportunity as well as entrepreneurial performance from a static perspective. However, when facing adversities, entrepreneurs often use pivoting as an important coping strategy [11]. Therefore, empirically investigating how entrepreneurial resilience influences entrepreneurs’ dynamic adjustment behavior toward entrepreneurial opportunities can provide a more comprehensive understanding of entrepreneurial resilience.

### 2.2. Resilience Diversity in Entrepreneurial Teams and Pivoting

Research on team resilience has essentially extrapolated individual average resilience to the team level. While this approach has been supported by research on team resilience and research on individual traits within teams [34], it introduces a dimension that has no parallel at the individual level—entrepreneurial resilience diversity, which has remained overlooked. However, substantial empirical studies indicate that diversity in demographics and psychological traits within teams may significantly affect team functioning and performance [23,29]. Therefore, this study focuses on the impact of resilience diversity on pivoting to develop a comprehensive understanding of the role of team resilience diversity in identifying and exploiting new entrepreneurial opportunities at the team level.

On the basis of the dynamic perspective of entrepreneurial opportunity, the pivot is a substantial and directional modification in the fundamental logic of original entrepreneurial opportunities by entrepreneurs after the establishment of new ventures. Driven by stakeholder feedback and external environmental changes, the risky and experimental adjustment of the original opportunity represents a complete departure from original entrepreneurial avenues in search of entirely new opportunities [4]. Limited research has explored the influence of resilience on pivoting and strategic changes. Such studies indicate that resilience is essential to the long-term survival of new ventures in turbulent environments because it enables entrepreneurs to enact strategic transformations [16,17] and to pivot by adjusting entrepreneurial opportunities [15].

Compared with the positive influence of average team resilience on strategic changes and the pivot [15], we propose that resilience diversity may negatively impact pivoting. Based on the study of team diversity, resilience diversity refers to the extent of variation in individual entrepreneurial resilience within a team. In contrast to the literature on functional heterogeneity among team members, which primarily investigates diverse work and industry experiences from the perspective of information advantages [23], this study focuses on deep-level diversity—entrepreneurial resilience diversity—which can be detrimental to entrepreneurial decisions, especially the pivot. As a resource-intensive entrepreneurial decision that involves prolonged commitment [50], pivoting is the activity of discovering new opportunities and reconfiguring critical resources to effectively exploit new opportunities [3]. Therefore, we will focus on the impact of the three dimensions of resilience diversity on discovering and exploiting new opportunities [22].

One’s recovering ability emphasizes entrepreneurs’ capability to promptly stabilize their emotions when confronted with adversity [51]. In a team with high resilience diversity, individuals with lower resilience may continuously experience negative emotions and anxiety, whereas those with higher resilience can proactively and quickly regulate their emotions. Such an incongruent emotional status may cultivate a tense and hostile atmosphere among team members, thus leading to relational conflicts [52]. In terms of opportunity discovery, the complex and challenging process of the pivot requires team members to concentrate on collecting and exploring information that is related to new opportunities. However, relational conflicts that arise from resilience diversity may distract team members’ attention from task-related information toward relational and emotional conflicts, which may diminish the team’s ability to identify new entrepreneurial ideas and opportunities [53]. In terms of opportunity exploitation, the divergence in emotional status that is induced by resilience diversity may amplify relational conflicts and erode trust and cohesion [54], which consequently disrupts the formation of consensus among team members during the pivoting process and hampers the exploitation of new opportunities.

One’s coping ability focuses on the entrepreneur’s capability to actively transform and overcome adversities [22]. We propose that, in the process of coping with crises, a high resilience diversity may lead to incongruence in acquiring and interpreting opportunity-related information when identifying new opportunities, and increase conflicts in opportunity exploitation strategies, which may be detrimental to the pivot. In terms of opportunity identification, entrepreneurs’ ability to discover new opportunities hinges on their cognition patterns and alertness [55]. However, entrepreneurs with different levels of resilience emphasize and interpret opportunity-related information in different ways [34]. On the one hand, in a team with high resilience diversity, highly resilient members are inclined to adapt to an ever-changing environment, quickly detect market opportunities, and foster innovation. Conversely, individuals with low resilience may adopt more conservative actions [56]. Therefore, high resilience diversity may increase the difficulty of reaching a consensus about highly risky and innovative new entrepreneurial opportunities, thereby hindering pivot decisions. On the other hand, the process of interpretating collective information depends on collaborative efforts for information exchange, discussion, and integration. Individuals may be less likely to understand each other’s viewpoints when team members with different levels of resilience emphasize different goals [32]. In turn, this lack of understanding may escalate team conflicts and thus hinder the identification of new entrepreneurial opportunities. In terms of opportunity exploitation, pivoting is a resource-consuming activity [50] because the core business will be changed to relatively unfamiliar domains. Therefore, during the pivoting process, entrepreneurial teams may lack the resources required for exploiting new opportunities, and team members may need to invest in human capital and build co-specialized assets [57]. However, differences in resilience within a team can lead to disparities in adaptive capabilities and objectives [32], which may induce conflicts in cognition and values [58]. These conflicts may affect the frequency and effectiveness of communication among team members and result in insufficient human capital investments. As a result, this issue impedes the effective process of opportunity exploitation that is necessary for pivoting. Furthermore, a high resilience diversity may generate more conflicts in resource allocation decisions. Pivoting requires entrepreneurs to reallocate resources from the original opportunity to develop entirely new ones [3]. Highly resilient entrepreneurs may invest abundant resources in transformative actions to survive adversity, which may not be endorsed by individuals with lower resilience [56]. This ultimately hampers the exploitation of new opportunities.

One’s thriving ability refers to entrepreneurs’ capability to learn and obtain growth from setbacks [7,22]. Given that the pivot entails structural adjustments to the core logic of original opportunities, it often results in organizational upheavals [5] or strategic repositioning [59], and thus requires a broader knowledge base. In order to make effective pivot decisions, entrepreneurs need to engage in entrepreneurial learning to conduct a cross-boundary search and update their knowledge across different markets. While highly resilient entrepreneurs are able to learn from adversity, sharing knowledge among team members is difficult when entrepreneurs with low resilience exhibit less proactiveness in learning from crises [8]. Therefore, high resilience diversity may cause teams to experience slower updates in their knowledge base, thus limiting their cross-boundary search activities [60]. This constraint reduces the likelihood of identifying unmet new market demands and subsequently hampers the pivot. Furthermore, individuals with lower resilience are more likely to exit the team, whereas those with higher resilience are more inclined to persist and engage in learning [32]. The diversity of entrepreneurial goals disrupts team communication, therefore influencing the collective interpretation process of newly acquired information through learning [34]. Hence, resilience diversity hinders the effectiveness of entrepreneurial learning and, subsequently, the identification and exploitation of new opportunities [61]. Therefore, we propose the following:

**Hypothesis** **1** **(H1).** *Resilience diversity in new venture teams negatively affects pivoting.*

### 2.3. Resilience Diversity and Team Reflexivity

Team reflexivity is the collective process through which team members publicly reflect on the team’s objectives, strategies, and procedures, and make adjustments based on internal and external environments [27,31]. Team reflexivity allows members to adapt to the changes and cope with uncertainties in the external environment. As a unification of cognitive and behavioral processes, team reflexivity includes three activities: reflection, planning, and action/adjustment [27]. Reflection refers to the thinking process of task-related problems, including team goals, strategies, processes, and the environment. Planning entails setting goals and making plans for achieving those goals, thus serving as a bridge between reflection and adaptive actions. Action/adjustment denotes the execution of plans to achieve anticipated changes [27]. Given its significance for team efficacy and innovation [31], team reflexivity has received considerable research attention.

However, team reflexivity rarely occurs spontaneously within teams [62]. One of the central topics of prior research has focused on how team characteristics promote team reflexivity. From the perspective of information acquisition, members with diverse functional backgrounds can provide heterogeneous information and viewpoints [23], which stimulates thinking and reflexivity processes [63]. Although the information acquisition perspective is not limited to functional diversity [64], scholars suggest that this perspective may be less applicable to deep-level social diversity [34], such as resilience diversity.

According to sensemaking theory, making sense of external events, such as adversities, involves the incorporation of different understandings into the team’s shared cognitive frameworks [65]. We propose that members with different levels of resilience have distinct cognitive patterns and values, which may lead to the selective processing and filtering of task-related information [56]. This effect can hinder in-depth collective reflexivity on work methods and task progress.

First, in the face of adversity, highly resilient members may strive to effectively regulate their emotions, while less resilient individuals have a worse recovering ability [51]. In teams with high resilience diversity, less resilient members may experience negative emotions, which may lead to evasive attitudes toward adversity, and hinder their ability to engage in high-quality interactions with other members and offer emotional support to the team [52]. Low-quality team interactions can impede the integration and coordination of cognitive resources among team members [66,67]. As a result, forming a shared understanding of tasks at the team level becomes challenging in teams with high resilience diversity, making it more difficult to engage in the sensemaking process to adjust the objectives, strategies, and processes. The existing diversity literature supports the theoretical logic that diversity in motivation and emotions may increase relational conflicts and hinder team members from sharing and integrating their information and thoughts [34]. Therefore, resilience diversity increases the barriers to team collaboration and integration in information exchange among members, and impedes the process of sensemaking through environmental cues, thereby hindering team reflexivity.

Second, a higher resilience diversity may lead to inconsistent entrepreneurial goals and coping strategies, which may harm the meaningful sensemaking process of team reflexivity. Sensemaking is a cyclic process that includes attention, interpretation, and action [35]. As a dynamic process of information search and interpretation, team reflexivity requires intensive and proactive interactions among members to communicate about tasks and challenges [63]. However, the motivation of team members to communicate information depends on the congruence of their goals [62]. Consistent goals provide a reference framework for team activities, which can foster positive interactions, motivate members to fully engage in sensemaking from different perspectives and viewpoints, and thus facilitate effective task reflexivity [25]. Resilience diversity leads to inconsistent entrepreneurial goals [32] and ways to interpret information among team members [34]. Therefore, members may be less likely to follow and understand each other’s opinions. This lack of cooperation obstructs further attempts to communicate [68], thereby reducing the motivation and accuracy of team reflexivity.

Third, high resilience diversity leads to different willingness and capabilities of the members to learn from experiences when facing adversity, and further harms teams’ ability to conduct effective reflexivity activities. Members with lower resilience are less likely to engage in learning-related activities [45]. However, according to the sensemaking theory, reflexivity activities require team members to integrate new understandings and opinions in the shared cognitive framework through collective learning activities [28]. Therefore, teams with high resilience diversity may face greater difficulties in collective learning activities and struggle to foster an atmosphere of questioning and discussing viewpoints [69]. As a result, entrepreneurial teams with significant diversity in resilience engage in fewer learning activities, thus making it difficult to make sense of chaotic, disorderly, and vague information into meaningful symbols and language [25]. This may reduce the accuracy of team reflexivity. Hence, we propose the following:

**Hypothesis** **2** **(H2).** *Resilience diversity in new venture teams negatively affects team reflexivity.*

### 2.4. The Mediating Effect of Team Reflexivity

Team reflexivity is essential for teams with complex and unconventional tasks in uncertain environments [31]. Therefore, team reflexivity is crucial in the pivot activities that involve high uncertainty and novelty. We focus on how resilience diversity affects the process of team reflexivity and therefore pivoting.

Many scholars have regarded team reflexivity as an antecedent of innovation and creativity [27]. For instance, West [70] emphasized that team reflexivity helps to foster a conducive atmosphere for the active discussion of innovative ideas in top management teams [71] and is an important factor of innovation. Dayan and Basarir [63] contended that reflexivity in product development teams helps generate diverse novel ideas and develop new products. In this study, we propose that the dual focus of team reflexivity [26], which entails the reflection on previous strategies and the preparation for future actions [72], is beneficial to pivoting, a strategic decision that involves abandoning the original opportunity and exploiting new ones [3,12].

First, entrepreneurs’ decisions to pivot are often enacted when they believe the current business is fundamentally not viable [5], which requires a systematic evaluation of the feasibility of the original opportunity. Team reflexivity encourages members to engage in systematic information processing and make adjustments based on the information [61]. During the process of team reflexivity, team members can thoroughly discuss various opinions, and effectively discover, question, and scrutinize the problems of the current business [73]. The systematic information processing of reflexivity aids teams in overcoming information biases [61], which reduces the escalation of commitment to the original opportunity, thereby making high-quality pivot decisions. In contrast, teams with low levels of reflexivity tend to disregard team plans, goals, and performance, and are more likely to take conservative actions [73]. Therefore, team reflexivity enables entrepreneurial teams to engage in the sensemaking activities of recognizing and interpreting problems and information that are relevant to the original opportunity, enhance the accuracy of opportunity evaluation and reduce path dependence bias, and therefore provide motivation for pivoting.

Moreover, pivoting requires not only the team’s recognition of the problems of the original opportunity but also the discovery and exploitation of a valuable new opportunity. By encouraging the questioning of current goals, team reflexivity stimulates members to express their opinions publicly, and fosters an organizational environment for members to update thinking patterns [65]. Thus, it encourages the discovery and exploitation of new opportunities. For opportunity discovery, team reflexivity enables teams to monitor signals and interpret them accurately from internal and external environments [74], thereby enhancing the team’s alertness to identify potential opportunities. Moreover, team reflexivity can stimulate positive interactions and information sharing, thus leading to the rational allocation and utilization of knowledge within the team [61]. This sensemaking activity helps to spark members’ creativity and enhance their ability to discover new opportunities. Prior research has indicated that teams engaging in collective reflexivity activities are more likely to experience the emergence of creative entrepreneurial ideas and develop innovative problem-solving solutions [63].

For opportunity exploitation, the pivot is a resource-intensive activity because it requires entrepreneurial teams to exploit an entirely new opportunity [50]. Team reflexivity encourages teams to take concrete action in implementing new entrepreneurial ideas [27]. First, teams that engage in numerous reflexivity activities are more likely to possess additional cognitive resources for making effective action plans, experience less psychological stress, and be more mentally and behaviorally prepared for change [74]. These teams can increase their members’ ability to rapidly adapt to an entirely new opportunity during the process of pivoting. Furthermore, team reflexivity aids team members in obtaining peer feedback and heterogeneous information, which enhances the frequency and accuracy of information sharing [61] and helps to formulate higher-quality plans and decisions. Reflective teams may critically evaluate new opportunities, abandon unpromising opportunities, and select better opportunities, which reduces the waste of resources during the exploitation process of a new opportunity. Therefore, after reflexivity activities, members can swiftly and accurately propose concrete implementation strategies for new opportunity exploitation during the pivot decision-making process. Based on the hypotheses in the previous sections, we propose the following:

**Hypothesis** **3** **(H3).** *Team reflexivity mediates the relationship between resilience diversity and pivoting.*

### 2.5. The Moderating Effect of Environmental Hostility

Environmental hostility refers to the individual’s perception of resource scarcity, market competitiveness, and difficulty in exploiting entrepreneurial opportunities [75]. On the one hand, the experience of adversity is an essential element in the formation and function of entrepreneurial resilience [22]. Environmental hostility reflects the extent to which a new venture faces unfavorable external conditions that hinder its operation and development, which may influence the manifestation of entrepreneurial resilience [30]. On the other hand, team reflexivity is a complex cognitive process that aims to help teams adapt to external environments [31]. In order to successfully engage in reflexivity activities, entrepreneurial teams need to closely monitor the changes in surrounding environments and adjust their actions accordingly. Therefore, the relationship between resilience diversity and team reflexivity may be moderated by environmental hostility.

In hostile environments, entrepreneurial teams may face intense competition, a challenging business atmosphere, and a lack of external resources [75]. Under such conditions, the negative effect of resilience diversity on team reflexivity is expected to be strengthened. First, in a hostile environment, entrepreneurial teams may face more extreme adversities, and the difference in the recovering abilities of members may be more severe. Therefore, environmental hostility may amplify the relational and emotional conflicts that arise from resilience diversity, thereby impeding the integration and collaborative utilization of cognitive resources for team reflexivity. Second, in a hostile environment, the information obtained by entrepreneurs is often complex and ambiguous [76]. Therefore, high resilience diversity may result in inconsistent information acquisition and interpretation among team members, thus leading to more severe decision conflicts. Furthermore, environmental hostility magnifies the incongruence in goals and values associated with resilience diversity. For instance, highly resilient entrepreneurs are more risk-taking, whereas less resilient entrepreneurs are more inclined to take conservative actions in more hostile environments [56]. The incongruence in coping strategies hinders the effective communication and discussion of viewpoints among team members, thereby hampering team reflexivity activities. Third, as for a thriving ability, highly hostile environments may be viewed as opportunities for learning by highly resilient entrepreneurs, while being interpreted as a signal to exit by those with low resilience [32]. In such environments, less resilient entrepreneurs are less likely to utilize environmental information and feedback to enhance their learning abilities, thereby hindering collective learning activities and team reflexivity. Therefore, in hostile environments, the negative effect of resilience diversity on team reflexivity is strengthened.

Combining the mediating effect hypothesis, we propose that environmental hostility moderates the mediation effect of team reflexivity on the relationship between resilience diversity and pivoting. First, in a hostile environment, strategic decisions are more complex and time-sensitive, thus requiring the effective allocation of the team’s attention resources [77]. Entrepreneurial teams need to increase cohesion and primarily focus on analyzing and solving task-related issues rather than coordination or relational problems. However, in a highly hostile environment, resilience diversity may lead to the excessive allocation of time and cognitive resources toward relational problems rather than task reflection activities [53], thereby diminishing the quality and commitment of the pivot decisions. Furthermore, when environmental hostility is high, the incongruence in entrepreneurial goals resulting from resilience diversity [32] also increases the difficulty for entrepreneurial teams to form a shared cognitive framework for effective reflexivity and learning [62]. This challenge harms the team’s ability to recognize the problems of original opportunities or discover new opportunities. Therefore, we propose that, in a highly hostile environment, the mediating effect of team reflexivity on the relationship between team resilience diversity and pivoting is strengthened. Based on these logics, we propose the following:

**Hypothesis** **4** **(H4).** *Environmental hostility moderates the negative effect of resilience diversity on team reflexivity, such that the negative effect is stronger in high environmental hostility.*

**Hypothesis** **5** **(H5).** *The mediating effect of team reflexivity is moderated by environmental hostility, such that the negative mediation is stronger in high environmental hostility.*

**Figure 1:**
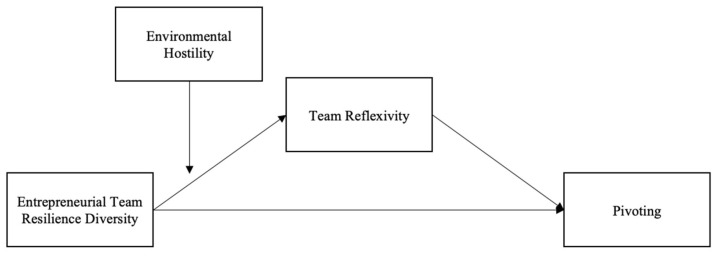
Theoretical model.

The theoretical model is demonstrated in Figure 1.

## 3. Methods

### 3.1. Participants

The analysis unit of this study is the entrepreneurial team. The data were collected through online and offline questionnaire surveys distributed to entrepreneurs in Chinese entrepreneurial incubators and MBA programs of a business school in China from December 2022 to October 2023. We recognized entrepreneurial team members by following three criteria: (1) holding shares of the entrepreneurial firm by investing financial capital or technological capital; (2) directly influencing the strategic decision-making process; and (3) devoting themselves to business management.

We collected survey data through two stages. The first stage data were used to test the reliability and validity of pivoting scale and 347 samples were obtained, with an effective sample rate of 69.4%. In the second stage, the two-wave data were used for hypothesis testing. In Wave 1, we collected data about entrepreneurial resilience, environmental hostility, and control variables, while in Wave 2, we collected data on team reflexivity and pivoting three months later. According to Li and Atuahene Gima [78], new ventures are firms established for less than 8 years. Therefore, we excluded samples for more than 8 years, samples with incomplete answers, and samples with less than 2 team members. In addition, in the questionnaire, entrepreneurs were asked to report whether they have discovered new opportunities that are different from the original opportunity during the entrepreneurial process, as well as a detailed description of the original opportunity and new opportunities. This study only included samples that discovered new opportunities different from the original ones. In the second stage, the final data were obtained from 426 entrepreneurs in 112 teams, with an effective sample rate of 85.2%.

Table 1 demonstrates the demographics of sample teams.

### 3.2. Measures

In this study, for latent variables, we employed the Likert seven-point scale and participants were asked to rate their agreement, ranging from “1” indicating “strongly disagree” to “7” indicating “strongly agree”. Given the Chinese research context, we translated from English scales to Chinese and back-translated the Chinese version to English to ensure the appropriateness of the Chinese questionnaire under the guidance and supervision of two professors in the entrepreneurship. Appendix A Table A1 shows the scales.

#### 3.2.1. Resilience Diversity

To measure entrepreneurial resilience diversity within a new venture team, we first used a six-item scale [6,42] to measure the entrepreneurial resilience of each member in the team. Then, we calculated the standard deviation of members’ resilience scores within a team to assess resilience diversity [79], which is widely used in prior studies on team diversity [24].

#### 3.2.2. Pivoting

Building on prior work on pivoting [3,80], we conducted a pilot study to develop a measurement scale of pivoting. Utilizing data from a pilot study, we performed EFA and CFA to assess the reliability and validity of the scale. EFA yielded a single factor accounting for 66.525% of the total variance and the factor loadings of all items are larger than 0.6. The model fit of CFA showed a good model fit (χ^2^/df = 1.245, GFI = 0.987, CFI = 0.997, TLI = 0.994, RMSEA = 0.035, SRMR = 0.0198). The Cronbach’s α = 0.864, CR = 0.8643, and AVE = 0.5607 provided evidence for good reliability and convergent validity. Furthermore, the correlation between pivoting and opportunity evaluation was positive (r = 0.273, *p* < 0.01), and the correlation between pivoting and entrepreneurial performance was negative (r = −0.157, *p* < 0.05). These results were consistent with prior studies, which demonstrated high criteria validity. In conclusion, a five-item scale was developed to measure pivoting, with an example item “We are committed to exploiting a new opportunity different from the original one”.

#### 3.2.3. Team Reflexivity

The five-item measurement of team reflexivity developed by De Jong and Elfring [72] and Carter and West [27] was employed in this study. A sample item included in the scale is: “Within the team, we frequently evaluate the feasibility of our goals”.

#### 3.2.4. Environmental Hostility

Environmental hostility was measured by a three-item scale [76], which captures the three perspectives of hostility: risks, opportunities, and market competition.

#### 3.2.5. Control Variables

Firm-level control variables were considered in this study, including the firm size, firm age, industry, and competition. Team-level variables included the average level of resilience and team size. Moreover, in order to reduce the confounding effect of other types of diversity, we controlled for the age diversity, education diversity, and gender diversity of the entrepreneurial team. Furthermore, we controlled for opportunity evaluation developed by Scheaf et al. [81], which has been proven to impact pivoting in prior studies.

## 4. Results

### 4.1. Reliability and Construct Validity

The CFA results revealed a good fit of the measurement model, as indicated by the following fit indices: χ^2^/df = 2.523, GFI = 0.923, CFI = 0.955, TLI = 0.947, RMSEA = 0.060, and SRMR = 0.0344. Table 2 presents the results of reliability and validity. For the measurement scale of entrepreneurial resilience, pivoting, team reflexivity, and environmental hostility, the Cronbach’s α, CR and AVE exceed the threshold of 0.7, 0.7, and 0.5, respectively, indicating good internal consistency and reliability. Furthermore, the square roots of AVE for all variables are greater than the correlation coefficients between variables, demonstrating the high discriminant validity of the scales.

### 4.2. Common Method Bias

In research design, we collected the data in two waves from team members. The multi-source and multi-wave research design can reduce the common method bias [82]. Moreover, we performed EFA and CFA to evaluate CMV problem. The first factor extracted in EFA explained 27.52% of the total variance of all items, below the threshold of 40%. The single-factor model in CFA resulted in bad model fit (χ^2^/df = 22.209 > 3, GFI = 0.451 < 0.9, CFI = 0.349 < 0.90, TLI = 0.267 < 0.90, RMSEA = 0.223 > 0.08, SRMR = 0.2258 > 0.08). These results indicate that CMV bias is not a major issue in our study.

### 4.3. Data Aggregation

As the entrepreneurial team is the unit of analysis for this study, we aggregated the data of all variables at the team level. We computed Rwg_(j)_, ICC (1), and ICC (2). In Table 3, Rwg_(j)_ and ICC (1) for four variables exceeds 0.70 and 0.05, and ICC (2) > ICC (1) [83], supporting the data aggregation of the four constructs.

### 4.4. Descriptive Statistics

The means, standard deviations, and correlation coefficients are displayed in Table 4. The correlation analysis revealed significant negative associations between resilience diversity and pivoting (r = −0.500, *p* < 0.01) and team reflexivity (r = −0.550, *p* < 0.01). These findings provide preliminary empirical support for H1 and H2.

### 4.5. Tests of Hypotheses

We used SPSS to perform OLS regressions to test our theoretical model and hypotheses. First, we calculated the VIF values. The VIF values of all variables were less than 10 and the average VIF is 1.39, suggesting that the problem of multicollinearity is not severe.

Table 5 demonstrates the results. The coefficient of resilience diversity on pivoting in Model 2 is significantly negative (*β* = −0.574, *p* < 0.01), showing strong evidence for H1. Interestingly, in contrast to the positive effect of average resilience in Model 1 (*β* = 0.219, *p* < 0.05), its positive coefficient is no longer significant in Model 2 (*β* = 0.085, *p* > 0.1), when adding resilience diversity into the regression model. These interesting results complement the previous studies which propose that the inclusion of highly resilient entrepreneurs into teams can increase the average resilience and be beneficial. Our results imply that introducing highly resilient entrepreneurs into teams may not always be beneficial because it may increase team diversity in teams with many low-resilience members and thus be detrimental to pivoting.

In Model 5, the result shows a negative relationship between resilience diversity and team reflexivity (*β* = −0.681, *p* < 0.01), which is consistent with H2. Also, the positive effect of average resilience in Model 4 (*β* = 0.236, *p* < 0.05) turns insignificant in Model 5 (*β* = 0.077, *p* > 0.1) when introducing the variable of resilience diversity.

We followed the method proposed by Baron and Kenny [84] to test the mediation effect of team reflexivity. In Model 3, when controlling the resilience diversity, the coefficient of team reflexivity on pivoting is significantly positive (*β* = 0.252, *p* < 0.01). Moreover, the absolute value of the coefficient of resilience diversity in Model 3 (*β* = −0.402, *p* < 0.01) is less than that in Model 2 (*β* = −0.574, *p* < 0.01). Furthermore, we utilized the PROCESS Macro to perform bootstrap tests. The result in Table 6 indicates that the 95% confidence interval [−0.3224, −0.0574] of the indirect effect does not include 0. Therefore, these results support the mediating effect of team reflexivity on the relationship between resilience diversity and pivoting, which is anticipated by H3.

**Figure 2:**
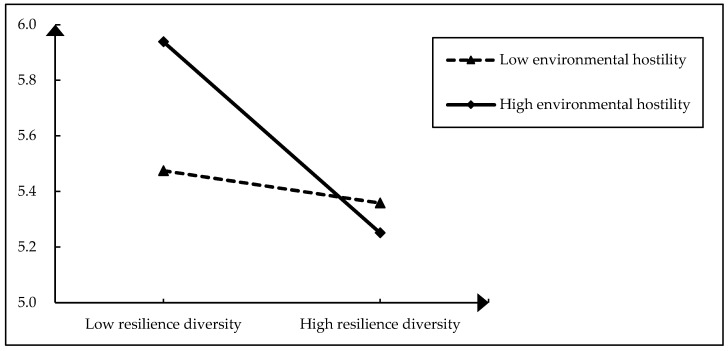
Moderating effect of environmental hostility.

Finally, we tested the moderation and mediated mediation. In Model 6 in Table 5, the interaction term of resilience diversity and environmental hostility is negatively correlated to team reflexivity (*β* = −0.643, *p* < 0.01), and the moderating effect in Figure 2 also supports H4. Furthermore, as for the moderated mediation, we performed bootstrap analysis in the PROCESS. As showed in Table 6, the index of a moderated mediation is significantly negative, and a 95% confidence interval also does not include 0 (index = −0.1623, 95% CI = [−0.3072, −0.0426]), supporting H5.

### 4.6. Robustness Checks

In this study, we conducted two robustness checks.

#### 4.6.1. Sample Adjustment

Prior studies have different definitions of new ventures. In the process of sample selection, we define a new venture as a company established within 8 years. However, other studies also suggest that a new venture is a company established within 6 years [85]. Therefore, we only retained 82 teams with their firm ages less than or equal to 6 years. The regression results are shown in Table 7, and all hypotheses are supported, indicating that the conclusion of this article is robust.

#### 4.6.2. Alternative Measurement and Econometric Model

Since the dependent variable of this study is pivoting, which is the entrepreneurial decision of the team, we use a dummy variable for alternative measurement. Previous studies have suggested that pivoting refers to entrepreneurs developing a new opportunity that is completely different from the original one. In the survey, we required entrepreneurs to report detailed descriptions of the original and new opportunities. Based on the specific information of the original and new opportunities (such as products, business models, etc.), we invited two professors and two doctoral students in entrepreneurship research to independently evaluate whether a team has made a pivoting decision. This method has been widely used by other scholars (such as Tost et al. [86]). As for the conflicts, we discussed the results to reach a consistent conclusion. We generated a dummy variable named pivot2 to indicate whether the team has taken a pivot decision. Pivot2 was coded 1 when the team pivoted, and 0 otherwise.

We used pivot2 to perform regressions and used the three-step method to test the whole model. Since pivot2 is a dummy variable and team reflexivity is a continuous variable, we used the logit regression model instead of multiple linear regression with pivot2 as the dependent variable, while we still used multiple linear regression with team reflexivity as the dependent variable [87]. Table 8 demonstrates the regression results. The results show that all hypotheses are still supported, indicating that the results of this article are robust.

## 5. Discussion and Conclusions

Traditional wisdom has reached a consensus that entrepreneurial resilience enables entrepreneurs to proactively embrace change in response to adversity [16,17], making them more inclined to adjust the original opportunity through pivoting [15]. However, the widely accepted assumption that entrepreneurial resilience is always beneficial may lead to a biased understanding of resilience [9]. This study focuses on the two research gaps that prior studies failed to address. First, previous studies have neglected the prevalence of team entrepreneurship [20]. The pivoting decisions are usually the result of consensus in entrepreneurial teams [5]. Therefore, the peer effect of the resilience level of team members may profoundly influence pivoting in the team context, which has been overlooked in prior research. Second, previous studies mainly investigated team resilience at the average level, assuming that team members have similar levels of resilience [21]. However, in entrepreneurial teams, individuals may have different levels of resilience [22]. Thus, the team entrepreneurship provides an interesting research context that emphasizes the variation and dispersion in resilience within teams, which has also been neglected in previous studies.

Hence, focusing on these research gaps, in this study, by introducing the concept of resilience diversity, we reveal the dark side of entrepreneurial resilience. Notably, grounded in sensemaking theory, we suggest that high resilience diversity within entrepreneurial teams can hinder their ability to pivot by hampering their recovering ability, coping ability and thriving ability. Moreover, we propose the underlying mechanism by which high resilience diversity reduces team reflexivity, and consequently diminishes the potential for pivoting. Furthermore, this dark side effect is strengthened in more hostile environments, such that the negative mediating effect of team reflexivity is stronger in high environmental hostility. The empirical results of 112 entrepreneurial teams provide support for this theoretical framework.

Moreover, in order to separate the effect of resilience diversity from average resilience, we deeply scrutinize and compare the two important properties of resilience in the team context. Surprisingly and interestingly, inconsistently with prior research, the beneficial effects of average resilience are no longer significant when we introduce the diversity of resilience into the regression model. This result provides important evidence that resilience diversity, rather than average resilience, is what influences teams’ reflexivity activities and pivoting decisions, in a negative way. Overall, our findings indicate that introducing highly resilient entrepreneurs into teams may not be always beneficial, especially in teams with many low-resilience members because it may increase team diversity and offsetting the benefits brought by average resilience, and finally harm pivoting.

### 5.1. Theoretical Contributions

Our study bridges the literature on entrepreneurial resilience, team diversity, and pivoting and provides significant theoretical implications.

First, previous research on entrepreneurial resilience has primarily focused on its positive effects as psychological capital. For instance, entrepreneurial resilience has been proven to be beneficial to entrepreneurial performance [47], entrepreneurial learning [7,22], and subject well-being [46]. However, the benefits of resilience may be overstated by the literature while the negative effects still remain underexplored. This study responds to the call by Williams et al. [9] and Hartmann et al. [32] for more research on the dark side of resilience. Based on the sensemaking theory, this study proposes and validates the negative impact of resilience diversity on team reflexivity and pivoting, thus enriching our understanding of the potential dark side effects of resilience. Moreover, in contrast to prior literature that mainly investigates the impact of resilience on individuals and organizations, our study introduces a team perspective to explore the influence of resilience diversity within teams on members’ cognitive processes, i.e., team reflexivity. These explorations expand our knowledge of the dark side of resilience and enhance our understanding of how resilience operates within teams.

Second, as the first empirical study to analyze resilience diversity at the team level, our study underscores the importance of investigating the structure of resilience. Building on the emerging body of research on team resilience [43], we explore a previously overlooked feature of resilience—the structure of resilience by introducing the concept of resilience diversity. This study links resilience as an individual trait and emotional characteristic to entrepreneurial team diversity [23]. Conceptually, we differentiate between average team resilience and resilience diversity, which capture different but equally important attributes of team resilience, namely the mean and dispersion properties, respectively. Empirically, the results of our study indicate that resilience diversity plays distinct roles in team cognition and the decision-making process. Interestingly, while simultaneously considering average resilience and resilience diversity, the positive effects of average resilience on reflexivity and pivoting become insignificant, and resilience diversity exerts a significantly negative impact. This exploration complements prior studies that focus on the average resilience and suggests that the inclusion of highly resilient entrepreneurs is beneficial [44] because it increases the average level of resilience within the team. In contrast, adding members with high resilience may increase resilience diversity when the team has many members with low resilience, which may harm reflexivity and pivoting. These findings indicate that the importance of examining the effects of team resilience at different levels of analysis should not be ignored, thereby making an important contribution to the literature on team resilience.

Third, while the research on pivoting is emerging, the existing literature on entrepreneurship still predominantly focuses on the identification and exploitation of original opportunities [10]. Existing research on pivot has examined how external antecedents, such as environmental conditions and stakeholder feedback, may drive entrepreneurs to pivot [3]. However, the internal factors of the pivot still lack investigation. Moreover, the literature focuses on individual entrepreneurs and overlooks the fact that team entrepreneurship has gradually become prevalent. This study focuses on a critical feature of entrepreneurial teams—resilience diversity—and explores its impact on the adjustment of the original opportunity, which provides a complementary research perspective to the literature on pivoting. Furthermore, research on pivoting mainly focuses on theoretical or case analysis [3], thus lacking large-sample empirical validation. Through a survey-based research design, this study increases the external validity and enriches the research method of pivoting.

Fourth, this study contributes to the literature on team reflexivity. Previous research on team reflexivity has neglected the effect of social diversity by mainly focusing on how team reflexivity is affected by its functional diversity, such as age diversity [73], gender, and educational diversity [62]. We emphasized the important impact of resilience diversity on team reflexivity, which is encouraged by Humphrey and Aime [33], to explore the effects of individual characteristics and their dispersion on team reflexivity. Additionally, this study bridges the literature on team reflexivity, a critical collective cognitive process, and pivoting. These explorations enrich the perspective of the antecedents of team reflexivity and expand our understanding of the effects and consequences of team reflexivity.

### 5.2. Practical Implications

Our study also has important practical implications for entrepreneurs.

First, the current research demonstrates the significance of the structure of resilience within entrepreneurial teams. While resilience is typically characterized as a positive trait, we suggest that entrepreneurs should also consider the negative outcomes of resilience in a team context. When forming entrepreneurial teams, entrepreneurs should consider the diversity in psychological traits [24], particularly resilience diversity, which may harm team reflexivity and the pivot decision. The insignificant coefficient of average resilience when adding resilience diversity into the model indicates that introducing highly resilient members into a team may not always be beneficial. It hinges on the resilience levels of other members. In general, this study provides practical guidance for entrepreneurs in the decision of team composition when introducing team members.

Moreover, the mediating effect of team reflexivity indicates its crucial role in the successful enactment of the pivot. Therefore, when facing crises and making pivot decisions, entrepreneurial teams should make full use of team reflexivity activities to collectively reflect on the team’s goals, strategies, and procedures [27,31] to make effective pivot decisions.

Finally, the boundary condition of environmental hostility demonstrates that selecting highly resilient entrepreneurs as team members in teams with many low-resilience members may increase resilience diversity, which is not necessarily advisable in extremely hostile environments. When the environment changes in an unfavorable direction, introducing some highly resilient entrepreneurs when the team is composed of many entrepreneurs with low resilience may amplify the negative effect of resilience diversity on the team’s ability to reflect and pivot, making it more difficult for the team to effectively respond to crises.

### 5.3. Limitations and Future Directions

Despite these contributions, this study has certain limitations that future research can address. First, although the current study explains the anticipated pitfalls of team resilience diversity, exploring ways to mitigate this dark side effect is valuable. In the future, scholars could investigate the influence of other potential moderating variables to alleviate the decline in team reflexivity caused by resilience diversity and provide more valuable guidance for entrepreneurial practice. Entrepreneurial team governance mechanisms, such as ownership concentration, may serve as important boundary conditions [88]. Appropriate team governance focuses on the reasonable allocation of rights and responsibilities among team members, which may mitigate the negative impacts of resilience diversity.

Second, this study only focused on the potential costs of entrepreneurial resilience in terms of pivoting and team reflexivity. Future research could explore the effects of resilience diversity on other variables. For instance, researchers could investigate how resilience diversity affects the quality of strategic decisions, commitment, and innovation within the firm, all of which are crucial to the development of startups.

Finally, the data in our study were collected through surveys. Although we collected the data through multiple sources and time points, and conducted several analyses, a common method bias may still exist. Actually, pivoting can occur at various stages of a startup’s development in the long run [3,10]. As such, the entire process from discovering to exploiting new opportunities by an entrepreneurial team requires longitudinal research to fully capture the complete trajectory of pivoting. Hence, future research may consider longitudinal designs or second-hand data to propose and test theoretical frameworks, to develop a more comprehensive understanding of pivoting.

### 5.4. Conclusions

This study challenges the widely accepted assumption that resilience is universally beneficial. Drawing from sensemaking theory, the current study introduces the concept of resilience diversity in the research context of team entrepreneurship and explores the negative effect of resilience diversity on team reflexivity and therefore pivoting. Using data from a two-wave survey of 112 entrepreneurial teams in China, this study finds that the resilience diversity negatively impacts pivoting, and team reflexivity mediates the relationship. Moreover, in more hostile environments, the mediating effect of team reflexivity is strengthened. We hope that these findings will inspire future research on the dark side effects of resilience by investigating other underlying mechanisms and boundary conditions. These endeavors may shed light on resilience and provide more valuable guidance for entrepreneurial practice.

## Figures and Tables

**Table 1:** Sample demographics.

Variable	Classification	Frequency	Percent (%)
Firm Size	Less than CNY 2 million	18	16.1
	CNY 2 million~CNY 10 million	35	31.3
	CNY 10 million~CNY 40 million	21	18.8
	CNY 40 million~CNY 400 million	29	25.9
	More than CNY 400 million	9	8.0
Firm Age	0~2 years	19	17.0
	2~4 years	25	22.3
	4~6 years	38	33.9
	6~8 years	30	26.8
Team Size	3 members	55	49.1
	4 members	35	31.3
	5 members	12	10.7
	6 members	9	8.0
	7 members	1	0.9
Industry	Internet industry	37	33.0
	Medical industry	14	12.5
	Manufacturing industry	24	21.4
	Electronic and computer industry	17	15.2
	Other industries	20	17.9

**Table 2:** Reliability and validity analysis.

Constructs	Items	Factor Loadings	Cronbach’s α	CR	AVE
Entrepreneurial resilience	ER1	0.783	0.907	0.910	0.627
	ER2	0.802			
	ER3	0.807			
	ER4	0.820			
	ER5	0.770			
	ER6	0.766			
Team reflexivity	TR1	0.746	0.919	0.917	0.688
	TR2	0.847			
	TR3	0.812			
	TR4	0.875			
	TR5	0.860			
Environmental hostility	EH1	0.816	0.852	0.848	0.650
	EH2	0.812			
	EH3	0.791			
Pivoting	P1	0.774	0.887	0.898	0.638
	P2	0.776			
	P3	0.817			
	P4	0.812			
	P5	0.814			

**Table 3:** Data aggregation analysis.

Constructs	Rwg_(j)_	ICC (1)	ICC (2)
Entrepreneurial resilience	0.940	0.283	0.600
Team reflexivity	0.964	0.450	0.757
Environmental hostility	0.815	0.555	0.826
Pivoting	0.974	0.492	0.787

**Table 4:** Descriptive statistics.

Variables	1	2	3	4	5	6	7	8	9	10	11	12	13	14
1. Resilience diversity	1													
2. Team reflexivity	−0.550 ***	1												
3. Environmental hostility	−0.487 ***	0.418 ***	1											
4. Pivoting	−0.500 ***	0.461 ***	0.285 ***	1										
5. Average resilience	−0.218 **	0.143	0.119	0.135	1									
6. Opportunity evaluation	−0.227 **	0.150	0.276 ***	0.339 ***	−0.082	1								
7. Gender diversity	0.166 *	−0.134	−0.250 ***	−0.219 **	−0.129	−0.292 ***	1							
8. Education diversity	0.093	−0.100	−0.073	−0.127	0.196 **	−0.008	0.091	1						
9. Age diversity	0.306 ***	−0.265 ***	−0.223 **	0.028	0.041	−0.238 **	−0.013	0.097	1					
10. Competition	0.056	0.044	−0.085	−0.027	−0.308 ***	−0.019	0.088	−0.017	−0.055	1				
11. Firm size	−0.184 *	0.010	0.030	0.122	0.278 ***	−0.017	0.005	0.174 *	0.019	−0.523 ***	1			
12. Industry	−0.034	0.078	−0.040	−0.024	−0.024	−0.003	0.145	0.135	−0.031	0.128	−0.145	1		
13. Firm age	0.094	−0.115	−0.158 *	−0.303 ***	−0.055	−0.235 **	0.096	0.007	0.193 **	0.090	−0.141	0.057	1	
14. Team size	0.237 **	−0.115	−0.184 *	−0.187 **	0.194 **	−0.124	0.131	−0.140	0.093	0.061	−0.125	−0.031	0.101	1
Mean	0.403	5.575	5.358	5.512	5.308	4.828	0.357	0.384	3.395	2.880	2.790	2.720	2.710	3.800
S.D.	0.322	0.440	0.691	0.409	0.440	0.451	0.175	0.203	2.257	0.941	1.226	1.502	1.045	0.985
VIF	1.944	1.562	1.497	-	1.483	1.296	1.243	1.234	1.221	1.525	1.602	1.095	1.131	1.285

Note: * *p* < 0.1, ** *p* < 0.05, *** *p* < 0.01.

**Table 5:** Regression results.

Variables	Pivoting			Team Reflexivity		
	Model 1	Model 2	Model 3	Model 4	Model 5	Model 6
Control Variables						
Average resilience	0.219 **	0.085	0.066	0.236 **	0.077	0.076
	(0.089)	(0.084)	(0.081)	(0.105)	(0.099)	(0.092)
Opportunity evaluation	0.286 ***	0.219 ***	0.221 ***	0.071	−0.008	−0.014
	(0.084)	(0.076)	(0.073)	(0.099)	(0.090)	(0.084)
Gender diversity	−0.100	−0.042	−0.012	−0.188	−0.118	−0.147
	(0.214)	(0.192)	(0.185)	(0.253)	(0.227)	(0.212)
Education diversity	−0.474 **	−0.288 *	−0.260	−0.333	−0.113	−0.114
	(0.181)	(0.166)	(0.160)	(0.213)	(0.196)	(0.181)
Age diversity	0.034 **	0.053 ***	0.058 ***	−0.042 **	−0.018	−0.015
	(0.016)	(0.015)	(0.014)	(0.019)	(0.017)	(0.016)
Competition	0.068	0.041	0.034	0.059	0.027	0.028
	(0.044)	(0.040)	(0.038)	(0.052)	(0.047)	(0.043)
Firm size	0.043	0.013	0.019	0.012	−0.023	−0.005
	(0.034)	(0.031)	(0.030)	(0.040)	(0.037)	(0.034)
Industry	0.008	−0.000	−0.005	0.028	0.018	0.031
	(0.024)	(0.021)	(0.020)	(0.028)	(0.025)	(0.023)
Firm age	−0.087 **	−0.096 ***	−0.090 ***	−0.014	−0.025	0.010
	(0.034)	(0.031)	(0.030)	(0.041)	(0.036)	(0.035)
Team size	−0.086 **	−0.037	−0.036	−0.063	−0.004	0.013
	(0.038)	(0.035)	(0.034)	(0.044)	(0.041)	(0.038)
Independent Variables						
Resilience diversity		−0.574 ***	−0.402 ***		−0.681 ***	−0.626 ***
		(0.113)	(0.122)		(0.133)	(0.133)
Team reflexivity			0.252 ***			
			(0.081)			
Environmental hostility						0.130 **
						(0.058)
Resilience diversity × environmental hostility						−0.643 ***
						(0.169)
Constant	3.299 ***	4.426 ***	3.002 ***	4.311 ***	5.648 ***	4.651 ***
	(0.732)	(0.692)	(0.808)	(0.864)	(0.815)	(0.823)
R^2^	0.300	0.445	0.494	0.160	0.336	0.442
Model F	4.34	7.28	8.04	1.93	4.59	5.98

Notes: standard errors in parentheses, * *p* < 0.1, ** *p* < 0.05, *** *p* < 0.01.

**Table 6:** Bootstrap results.

Mediation						
	Effect	SE	t	*p*	95% CI	
					LLCI	ULCI
Direct effect:						
Resilience diversity→pivoting	−0.4025	0.1215	−3.3119	0.0013	−0.6436	−0.1613
Indirect effect:						
RD→team reflexivity→pivoting	−0.1717	0.0679			−0.3224	−0.0574
**Conditional indirect effect**						
**Mediator**	**Moderator**	**Effect**	**SE**		**95% CI**	
					**LLCI**	**ULCI**
Team reflexivity	−0.6906	−0.0457	0.0511		−0.1671	0.0327
Team reflexivity	0.0000	−0.1578	0.0641		−0.3026	−0.0528
Team reflexivity	0.6906	−0.2698	0.0995		−0.4818	−0.0937

**Table 7:** Robustness check results of sample adjustment (N = 82 teams).

Variables	Pivoting			Team Reflexivity		
	Model 1	Model 2	Model 3	Model 4	Model 5	Model 6
Control Variables						
Average resilience	0.266 **	0.169	0.141	0.204	0.103	0.043
	(0.124)	(0.110)	(0.106)	(0.131)	(0.117)	(0.098)
Opportunity evaluation	0.327 ***	0.254 ***	0.250 ***	0.090	0.014	0.021
	(0.100)	(0.089)	(0.085)	(0.106)	(0.095)	(0.079)
Gender diversity	−0.126	0.066	0.110	−0.358	−0.158	−0.249
	(0.273)	(0.243)	(0.234)	(0.290)	(0.259)	(0.216)
Education diversity	−0.521 **	−0.356	−0.355 *	−0.175	−0.002	−0.056
	(0.241)	(0.214)	(0.206)	(0.256)	(0.228)	(0.190)
Age diversity	0.047 **	0.075 ***	0.083 ***	−0.056 **	−0.027	−0.021
	(0.023)	(0.021)	(0.020)	(0.024)	(0.022)	(0.018)
Competition	0.076	0.052	0.040	0.069	0.045	0.057
	(0.055)	(0.048)	(0.047)	(0.058)	(0.052)	(0.043)
Firm size	0.046	0.001	0.012	0.009	−0.037	0.014
	(0.043)	(0.039)	(0.038)	(0.046)	(0.041)	(0.036)
Industry	−0.011	−0.023	−0.019	−0.001	−0.013	−0.001
	(0.031)	(0.027)	(0.026)	(0.033)	(0.029)	(0.025)
Firm age	−0.114 **	−0.127 **	−0.112 **	−0.040	−0.053	−0.021
	(0.057)	(0.050)	(0.048)	(0.061)	(0.053)	(0.045)
Team size	−0.092 *	−0.045	−0.047	−0.040	0.008	0.070 *
	(0.051)	(0.046)	(0.044)	(0.054)	(0.049)	(0.042)
Independent Variables						
Resilience diversity		−0.651 ***	−0.461 ***		−0.679 ***	−0.452 ***
		(0.136)	(0.150)		(0.145)	(0.139)
Team reflexivity			0.279 **			
			(0.108)			
Environmental hostility						0.273 ***
						(0.068)
Resilience diversity × environmental hostility						−0.823 ***
						(0.185)
Constant	2.920 ***	3.905 ***	2.376 **	4.453 ***	5.480 ***	3.608 ***
	(0.938)	(0.846)	(1.005)	(0.995)	(0.902)	(0.842)
R^2^	0.324	0.490	0.535	0.211	0.399	0.596
Model F	3.41	6.12	6.63	1.90	4.22	7.72

Notes: standard errors in parentheses, * *p* < 0.1, ** *p* < 0.05, *** *p* < 0.01.

**Table 8:** Robustness check results of alternative measurement and econometric model.

Variables	Pivot2			Team Reflexivity		
	Model 1	Model 2	Model 3	Model 4	Model 5	Model 6
Control Variables						
Average resilience	0.754 **	0.350	0.386	0.236 **	0.077	0.076
	(0.369)	(0.388)	(0.452)	(0.105)	(0.099)	(0.092)
Opportunity evaluation	0.749 **	0.618 *	0.856 **	0.071	−0.008	−0.014
	(0.303)	(0.322)	(0.345)	(0.099)	(0.090)	(0.084)
Gender diversity	0.465	1.082	2.015 *	−0.188	−0.118	−0.147
	(0.878)	(0.918)	(1.082)	(0.253)	(0.227)	(0.212)
Education diversity	−2.124 ***	−1.708 **	−2.208 ***	−0.333	−0.113	−0.114
	(0.713)	(0.777)	(0.745)	(0.213)	(0.196)	(0.181)
Age diversity	0.043	0.151 **	0.265 ***	−0.042 **	−0.018	−0.015
	(0.060)	(0.063)	(0.076)	(0.019)	(0.017)	(0.016)
Competition	0.104	−0.013	−0.101	0.059	0.027	0.028
	(0.165)	(0.158)	(0.179)	(0.052)	(0.047)	(0.043)
Firm size	0.151	0.045	0.011	0.012	−0.023	−0.005
	(0.118)	(0.126)	(0.151)	(0.040)	(0.037)	(0.034)
Industry	0.044	0.006	−0.025	0.028	0.018	0.031
	(0.093)	(0.099)	(0.114)	(0.028)	(0.025)	(0.023)
Firm age	−0.209	−0.307 **	−0.470 **	−0.014	−0.025	0.010
	(0.128)	(0.144)	(0.183)	(0.041)	(0.036)	(0.035)
Team size	−0.127	0.066	0.115	−0.063	−0.004	0.013
	(0.135)	(0.152)	(0.164)	(0.044)	(0.041)	(0.038)
Independent Variables						
Resilience diversity		−0.574 ***	−0.402 ***		−0.681 ***	−0.626 ***
		(0.113)	(0.122)		(0.133)	(0.133)
Team reflexivity			0.252 ***			
			(0.081)			
Environmental hostility						0.130 **
						(0.058)
Resilience diversity × environmental hostility						−0.643 ***
						(0.169)
Constant	−6.695 **	−3.448	−16.953 ***	4.311 ***	5.648 ***	4.651 ***
	(2.801)	(2.947)	(4.692)	(0.864)	(0.815)	(0.823)
Pseudo R^2^/R^2^	0.145	0.279	0.419	0.160	0.336	0.442
Wald chi^2^/Model F	24.38	49.69	38.12	1.93	4.59	5.98

Notes: standard errors in parentheses, * *p* < 0.1, ** *p* < 0.05, *** *p* < 0.01.

## Data Availability

Data available upon request from the authors.

## Appendix A

**Table A1:** Measurement scales.

Constructs	Items	Measurement Items	References
Entrepreneurial resilience	ER1	1. When I have a setback at work, I have trouble recovering from it, moving on. (R)	Luthans et al. [6], Haddoud et al. [42]
	ER2	2. I usually manage difficulties one way or another at work.	
	ER3	3. I can be ‘‘on my own,’’ so to speak, at work if I have to.	
	ER4	4. I usually take stressful things at work in stride.	
	ER5	5. I can get through difficult times at work because I’ve experienced difficulty before.	
	ER6	6. I feel I can handle many things at a time at this job.	
Pivoting	P1	We are committed to developing new opportunities distinct from the original opportunity.	Kirtley and O’Mahony [3], Wood et al. [80]
	P2	2. We fundamentally changed the initial business logic and practices.	
	P3	3. We are turning to a brand-new opportunity.	
	P4	4. We are committed to replacing existing products/services/technologies or business with new ones.	
	P5	5. Our core products/services/technology or business are completely different from the past.	
Team reflexivity	TR1	In this team we often review the feasibility of our objectives.	De Jong and Elfring [72], Carter and West [27]
	TR2	2. In this team we often discuss the methods used to get the job done.	
	TR3	3. In this team we regularly discuss whether we are working effectively together.	
	TR4	4. In this team we modify our objectives in light of changing circumstances.	
	TR5	5. In our team we often review our approach to getting the job done.	
Environmental hostility	EH1	The external environment is very risky, and a false step can mean my firm’s undoing.	Covin and Slevin [76]
	EH2	2. The external environment is very stressful, exacting, hostile; very hard to keep afloat.	
	EH3	3. My firm is operating in a dominating environment in which my firm’s initiatives count for very little against the tremendous competitive, political, or technological forces.	
